# Poor oral habits and malocclusions after usage of orthodontic pacifiers: an observational study on 3–5 years old children

**DOI:** 10.1186/s12887-019-1668-3

**Published:** 2019-08-22

**Authors:** Silvia Caruso, Alessandro Nota, Atanaz Darvizeh, Marco Severino, Roberto Gatto, Simona Tecco

**Affiliations:** 1 Department of Life, Health and Environmental Science (MeSVA), University of L’Aquila, Piazzale Salvatore Tommasi 1, Blocco 11, 67100 L’Aquila, Coppito Italy; 2 Dental School, Vita-Salute San Raffaele University and IRCCS San Raffaele Hospital, via Olgettina 58, 20132 Milan, Italy

**Keywords:** Fingersucking, Thumbsucking, Pacifiers, Malocclusions, Tongue habits, Oral habits

## Abstract

**Background:**

Pacifier sucking habit has been associated in the literature with alterations of dental occlusion, and it could be a predisposing factor for other poor oral habits among children. Orthodontic pacifiers have been introduced in the market aiming to reduce these disadvantages caused by the conventional type of pacifiers. The aim of this study was to evaluate the prevalence of poor oral habits and malocclusions, after usage of orthodontic pacifiers in children with primary dentition.

**Methods:**

A sample of 198 pre-school children, aged 3–5 years, (96 males and 102 females) who had exclusively used an orthodontic pacifier were included in order to assess the level of poor oral habits and the absence/presence of dental malocclusion. Firstly, children’s parents/legal guardians were given a validated questionnaire, then the children were clinically examined at a dental clinic.

**Results:**

Most of the children (79.79%) had started using the orthodontic pacifier within the first 3 months of life, and the 43.49% of them continued using it over a period of 2 years. The recorded percentage for those who had used it throughout sleep was 89.39%. Mouth breathing during the night was reported for 36.04% of the children. Tongue thrust swallow affected 16.16% of the sample. The 5.56% of the data indicated the presence of fingersucking/thumbsucking habit. The noted percentages for children with lip biting, lingual interposition between teeth at rest and those with nail biting, were 5.56, 12.63 and 15.15%, respectively. The regression revealed a significant contribution between early start of using an orthodontic pacifier with the prevalence of fingersucking/thumbsucking (OR 0.13, 95% CI 0.04–0.47, *p* = 0.0004).

This also reported a noticeable increase of the malocclusion prevalence among the female gender (OR 2.74, 95% CI 1.42–5.31), as well as those who were not exclusively breastfed (OR 2.26, 95% CI 1.17–4.37).

**Conclusions:**

Orthodontic pacifiers does not favor the development of poor oral habits, even if it has been used for a period of 2 years in children with primary dentition. Children who begin to use orthodontic pacifier between 0 and 3 months, are less likely to acquire fingersucking/thumbsucking habit. The use of an orthodontic pacifier appears not to be correlated with the prevalence of malocclusion in primary dentition, differently from what stated in literature about the conventional type of pacifier.

## Background

The use of pacifiers is accepted during the first year of life, because it decreases the risk of sudden infant death syndrome, due to its influences on autonomic and cardiovascular control; in addition, it could help to calm the children and improve his/her psychological development [1]. Also other non-nutritive habits such as thumbsucking or fingersucking are often implemented by infants/children to pacify and comfort themselves, as sucking is a natural instinct for a baby, and it is the baby’s first coordinated muscular activity [1]. However, from the craniofacial development point of view, the use of a conventional pacifier for a long time (over 2 years) [2] and with high frequency (a “daily use”, as recently stated by Ling et al. [1]) has been associated with some alterations of the occlusion, such as anterior open bite and posterior crossbite [3–5].

For this reason, the so-called orthodontic pacifiers have been introduced into the market, designed with a flattened nipple to simulate mothers’ nipple anatomy, to maintain the necessary pressure of tongue on the palatal vault and to obtain a more acceptable lip seal, allowing its physiological development and reducing the side effects related to utilising conventional pacifiers [6–9]. In fact, the use of such pacifiers would rise to replicate the patterns of muscle contraction, tongue position and nasal breathing similar to the ones occurring during breastfeeding, whereby they would not interfere with the growth and development of the face and occlusion [10].

The first systematic literature review on the differences between conventional and orthodontic pacifiers [11], was not able to draw any conclusion due to the low level of available studies, requesting more data on this field. That systematic review did not include parameters such as frequency and duration of pacifier usage. Another recent systematic review [12] including five trials about the comparison between orthodontic and conventional pacifiers [6, 7, 10, 13, 14] concludes that a proper definition for a functional or orthodontic pacifier is missing, and that functional orthodontic pacifiers seem to cause less anterior open bite than conventional ones, while the no statistical difference implies the absence of an association between the prevalence of posterior crossbite and the use of orthodontic pacifiers [6, 10]. Thus, the main conclusion stated the insufficiency of currently available evidence to support this hypothesis that the usage of orthodontic pacifiers is able to prevent malocclusion traits when compared to conventional ones, and that new data on orthodontic pacifiers effects are necessary in literature to substantiate the argument [8].

Orthodontic pacifiers, while reducing the occurrence of malocclusions, also lessen the risk of gaining those poor oral habits, which are harder to stop in children, for example fingersucking/thumbsucking. In other words, the rationale is that if the child gets the maximum satisfaction from orthodontic pacifier sucking (a non-dangerous sucking), he will not feel the need to acquire other poor habits. In addition, as an orthodontic pacifier can enhance the coordination between breathing and sucking-swallowing by oral stimulation, it could also prevent mouth breathing.

As poor oral habits and mouth breathing may be predisposing factors for formation of malocclusion, [4, 15, 16] knowledge of orthodontic pacifiers role to contribute or prevents them, could help in determining better options for children’s oral health care. But unfortunately, literature still lacks data on the frequency of poor oral habits and breathing pattern among those children using orthodontic pacifiers.

Thus, the aim of this observational study was to investigate the prevalence of poor oral habits and malocclusions, in children using an orthodontic pacifier.

## Methods

In this observational study, conducted at the University of l’Aquila (Central Italy), including a database of customers who gave their consent to Philips S.p.A. (Viale Sarca 235, 20,126 Milano, Italy) to participate in this screening event. The collected data covers purely a group of pre-school children aged 3–5 years, who had exclusively used the orthodontic pacifier brand called Philips Avent (Philips S.p.A., Viale Sarca 235, 20,126 Milano, Italy). Then, the parents/legal guardians have been contacted by an Orthodontics residency student from the University of L’Aquila, in order to provide them all the information regarding the research protocol. A free clinical oral examination at the dental clinic of the University was offered to all the contacted parents/legal guardians so as to encourage their participation in the study. The protocol agrees with the declaration of Helsinki and was approved by the ethical committee of the University of L’Aquila and the content validity of the questionnaire has been obtained in a qualitative way. In order to validate the questionnaire, a number of 15 experts in the field of orthodontics have been provided with the question sheet, to evaluate and comment on the grammar, use of proper words, transparency and correct placement of each question and its related answer options. Finally, according to the experts’ comments and suggestions, the questions were reconsidered and corrected. Then, the final edited version of the questionnaire was used to proceed with the present study.

The sample size was corresponding to a previously fulfilled cross-sectional study on the same topic, [17] in which the prevalence of malocclusion of 50%, a 95% confidence interval, and a standard error of 7% were assumed. The sample size of Lopes Freire et al. included a minimum of 195 children. Thus, during the initial stage a total number of 250 children were contacted.

About 210 of parents/legal guardians confirmed the participation of their children to the study, and a dental visit appointment has been scheduled for each participant. An informed consent form regarding the study and the dental examination has been handed to the parents at the beginning of the appointment, and it has been signed before clinical examination was carried out. Then, each parents/legal guardian has been provided with a validated questionnaire designed to collect information about the oral habits and general behaviors concerning oral health of their children and they utmost cooperation and honesty were requested while answering the questions. They were also asked not to hesitate to request any clarification in case of unclear questions. Lastly, the children were clinically examined on the dental chair, to determine the presence/absence of malocclusion and crossbite [18].

Tongue thrust swallow, tongue interposition between the dental arches at rest and tongue tie were also evaluated following a clinical protocol [19].

The clinical examination was performed by an experienced specialized orthodontist (S.C.), a principal investigator, with more than 5 years of orthodontic training and blind to the answers given to the questionnaire by the parents/legal guardians. The clinical examination of 15 children was repeated twice after a period of 15–30 days from the first examination, to evaluate the intra-operator method error and to assure that both examinations data agreed for 100% of the variables for all the children.

After the examination, to maintain the integrity of the study results, data from the assessed participants with severe skeletal discrepancy or craniofacial anomalies as cleft lip or palate, were excluded from the present analyses. In addition, also data from subjects with alterations of number, size and shape of deciduous teeth, or with major tooth destruction/reconstruction, systemic diseases and/or neurological diseases were excluded from analyses.

Therefore, data from a final sample of 198 children aged 3–5 years, with primary dentition were finally included in the present investigation. Ultimately a final data representing a sample of 198 children aged 3–5 years, with primary dentition were included in the present investigation.

### Statistical analyses

The Kolmogorov-Smirnov test was used to realize whether the data is normally distributed. The result of this analysis showed that the data was not normally distributed (*p*-value = 0.00). Hosmer & Lemeshow, VIF (Variance Inflation Factor) and Cook’s distance tests were utilized independently to measure the goodness-of-fit of this logistic model, using R software (3.5.1 version).

A descriptive statistical analysis was conducted to illustrate the characteristics of the sample, the data obtained from the questionnaires, the prevalence of poor oral habits and malocclusions. In order to study the relationship between orthodontic pacifier sucking, poor oral habits and malocclusion, cross-tabulation analysis was performed among variables and the χ2 test was used, providing Odds Ratios (OR) with a 95% confidence interval (CI) for each significant association.

Then the statistically significant variables were introduced to this logistic regression model, with the presence/absence of malocclusion and poor oral habits, placed as dependent variables.

It has been analyzed to verify whether resulted fitted to the data.

For each analysis, the threshold for statistical significance was set at *p* < 0.05.

## Results

Table 1 reports the characteristics and all the results of the present survey. As seen in Table 1, it emerged that most of the children (79.79%, 156 children out of 198) started using the orthodontic pacifier early, within the first 3 months of life, and often continued using it for a duration over 2 years. Thus, a great part of sample including 43.94% used the orthodontic pacifier for more than 2 years, while a lower percentage of children 31.82% used the pacifier for a period up to 2 years. The percentages of children who gladly used their orthodontic pacifier (78.28%, 155 children out of 198) and those who used it during sleep in the first year of life (89.39%, 177 children out of 198) were also very high.

**Table 1:** Descriptive characteristics of the sample

Variable	*n* (%)
Gender	
Male	96 (48.48)
Female	102 (51.52)
Age	
(in years)	3–5 years
What age did your baby start to use pacifiers at?	
0–3 months	156 (78.79)
4–6 months	26 (13.13)
7–12 months	16 (8.08)
How long did your baby use pacifiers?	
6 months	26 (13.13)
1 years	22 (11.11)
2 years	63 (31.82)
> 2 years	87 (43.94)
Did your baby start to use pacifiers gladly?	
No	43 (21.72)
Yes	155 (78.28)
Did your baby use the pacifier during sleep during the first year of life?	
No	21 (10.61)
Yes	177 (89.39)
Have you been informed at baby’s birth about how to make your baby sleep safely?	
Yes	181 (91.41)
No	17 (8.59)
Did your baby suffer recurrent otitis complaints?	
No	178 (89.90)
Yes	20 (10.10)
Was your baby exclusively breastfed for the first six months of life?	
Yes	132 (66.67)
No	66 (33.33)
Have you been informed at birth about benefits and not using pacifiers?	
Yes	111 (56.06)
No	87 (43.94)
Did your baby suffer gastro-oesophageal reflux problems (frequent regurgitation, vomiting after meals)?	
No	161 (81.31)
Yes	37 (18.69)
Does your child sleep with open mouth?	
No	126 (63.96)
Yes	71 (36.04)
Does your child suck his/her finger/thumb?	
No	187 (94.44)
Yes	11 (5.56)
Did your baby suffer with weaning?	
No	191 (97.46)
Yes	5 (2.54)
Does the child bite his/her lip?	
No	187 (94.44)
Yes	11 (5.56)
Does the child bite his/her nails?	
No	168 (84.85)
Yes	30 (15.15)
Clinical examination: presence of malocclusion	
No	127 (64.14)
Yes	71 (35.86)
Clinical examination: presence of crossbite	
No	162 (81.82)
Unilateral	28 (14.14)
Bilateral	8 (4.04)
Clinical examination: presence of tongue thrust swallow	
No	166 (83.84)
Yes	32 (16.16)
Clinical examination: tongue interposition between dental arches at rest	
No	173 (87.37)
Yes	25 (12.63)
Tongue tie	
No	178 (89.89)
Yes	20 (10.11)

When the sample was divided into two groups, based on the presence/absence of a malocclusion, compared to each other, statistically significant differences for gender distribution, with a significantly higher percentage of females with malocclusions compared to the males (63.38% vs 44.88%, *p* = 0.012), and for breastfeeding, which interested a lower proportion of children in the malocclusion group (71.65% vs. 57.75%, *p* = 0.046), were detected. The estimates of the ORs - adjusted for the effects of the other factors - are presented in the model (Table 2), through a logistic regression, that revealed a significant contribution of female gender (OR 2.74, 95% CI 1.42–5.31), and the not exclusive breastfeeding (OR 2.26, 95% CI 1.17–4.37) in increasing the probability of developing a malocclusion.

**Table 2:** Logistic regression analysis for the development of malocclusion and the explanatory variables (gender, breastfeeding)

Risk factor	OR°	95% CI
Gender		
Males^#^	1	
Females	2.74	(1.42–5.31) *
Was your baby exclusively breastfed for the first six months of life?		
Yes^#^	1	(1.17–4.37) *
No	2.26	

95% *CI* 95% confidence interval

^#^Reference category

*Statistically significant association

° adjusted ORs for the other factors in the model

The regression revealed a significant contribution of the beginning of orthodontic pacifier sucking on the prevalence of fingersucking/thumbsucking, because children who began to use orthodontic pacifier very early, between 0 and 3 months, were less likely to develop fingersucking/thumbsucking respect to children who begun after 3 months (OR 0.13, 95% CI 0.04–0.47, *p* = 0.0004) (Table 3).

**Table 3:** Logistic regression analysis for the association between development of fingersucking/thumbsucking and the age of beginning of orthodontic pacifier sucking)

Risk factor	OR°	95% CI
Age of beginning of orthodontic pacifier use:		
> 3 months^#^	1	
0–3 months	0.13	(0.04–0.47) *

95% *CI* 95% confidence interval

^#^Reference category

*Statistically significant association

° adjusted ORs for the other factors in the model

**Fig. 1:**
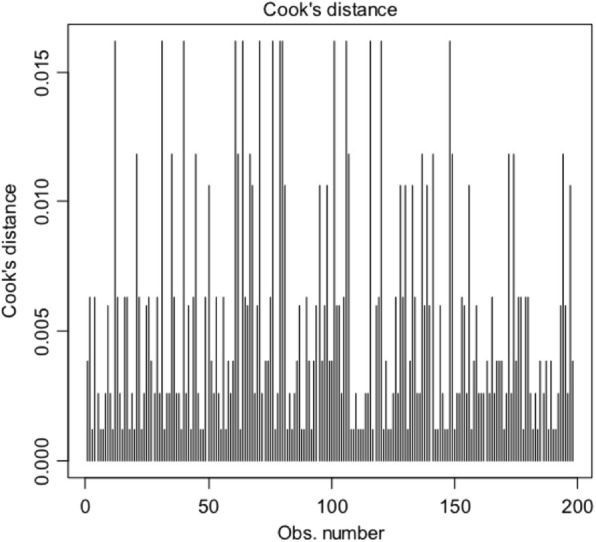
Cook’s distance plot to find influential outliers for model 1, the development of malocclusion and the explanatory variables (gender, breastfeeding)

For the model stated in Table 2, the Hosmer and Lemeshow goodness of fit test become non-significant since *P*-value = 1 (X-squared = 2.9107e-23, df = 2). To identify influential outliers, plot of Cook’s distance is shown in Fig. 1. There is no influential outliers because of all observation has the Cook’s distance less than 1. VIF for two predictors Gender, and Breastfeeding is 1.033. So we see from the output of the VIF function, that the variables Gender and Breastfeeding are not collinear.

**Fig. 2:**
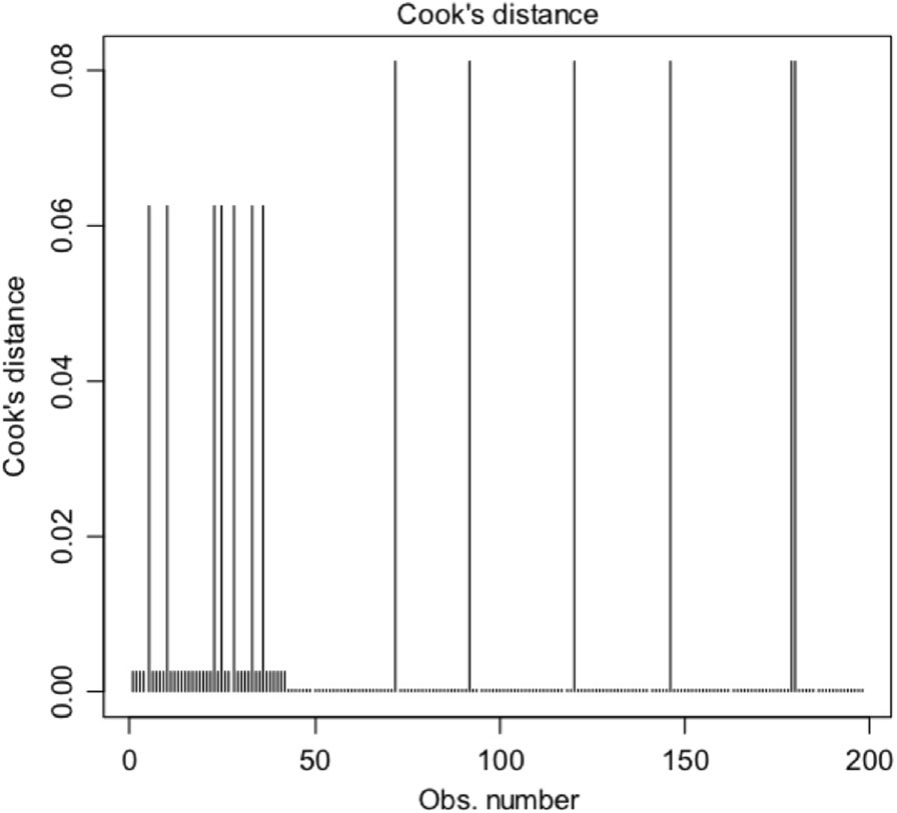
Cook’s distance plot to find influential outliers for model 2 (development of fingersucking/thumbsucking and the variable age of beginning of orthodontic pacifier sucking)

For the model stated in Table 3, Hosmer and Lemeshow test with *p*-value = 1 (X-squared = 3.045e-27, df = 2) suggests a good fit for the data. Further, since all points for Cook’s distance is less than 1, we can conclude that there is no influential outliers (see Fig. 2). Hence two models adequately describe the data.

No other associations were detected among the poor habits and the orthodontic pacifier sucking.

## Discussion

This observational study aimed to evaluate the prevalence of poor oral habits and malocclusions among children after using an orthodontic type of pacifier. The sample (198 children, ranging between 3 and 5 years of age) is undoubtedly the most extensive report among the published literature, recording the data of children who have exclusively used an orthodontic pacifier. Thus, the collected data can be generalized for the population of children who use orthodontic pacifiers. In addition, previously, no study has looked into whether or how orthodontic pacifier sucking is interrelated with poor oral habits, mouth breathing and tongue thrust swallow.

In the present sample, the great part of children (78.28% of the whole sample) gladly started utilizing orthodontic pacifier during their first 3 months of life (78.79% of the whole sample), adopting it regularly throughout the night, in their first year of age (89.39% of the whole sample). Thus, the present data can confirm that orthodontic pacifier was generally well accepted by children, as this fact has already been reported in various literatures [6, 10, 20]. In addition, the collected data of the present study revealed that the 91.41% of the parent/guardians had been properly informed right from the beginning about the advantages of orthodontic pacifiers over the conventional types and also the risks related to its prolonged usage. This also has been outlined that a great number of children were given their pacifiers during the night (89.39% of the whole sample), following the recommendations of pacifier usage, i.e. that it should be used while the infant is sleeping and not reinserted if the child drops it during sleep [21] (today, the recommended usage would be for sleeping and for less than 4–6 h per day) [22]. These data are in close agreement with the fact that an orthodontic pacifier is adopted by those mothers who are adequately informed concerning the risks of using conventional pacifiers. The present data also indicate that no significant correlation was detected between the duration of the pacifier sucking and the prevalence of recurrent otitis, as recurrent otitis was recorded only for 20 children out of 198 (10.10%). Differently from another previous report, which observed that pacifier sucking for a period exceeding 10 months, increases the risk of recurrent otitis [23].

Mouth breathing during the night was present in 71 children out of 198 (36.04% of the sample). The result of present study suggests that there exists a predominant pattern of nasal breathing among participated children. While, no relationship can be deduced between the breathing pattern and orthodontic pacifier sucking.

This result agrees with a recent survey from a sample of 1405 children between the ages of 2 and 7, (699 females and 706 males), in which oral breathing was present in 26.3% of the total children, concluding for the great benefits of an early interceptive treatment, as it is categorized as grade 2 of risk, requesting a periodical surveillance, and ear, nose, and throat evaluation for breathing problems. [24]

In a previous sample of 36 pre-school children with primary dentition using conventional pacifier, Nihi et al. found that 22.2% of them (8 subjects out of 36) had mouth breathing at rest, while only 8.3% (4 children out of 48) of pacifier non-users showed mouth breathing [2]. According to Nihi et al. a higher prevalence of mouth breathing among pacifier users with respect to controls, may be associated to the altered position of the tongue in the mouth, which causes these subjects to keep their mouths open and consequently develop a mouth-breathing pattern. This explanation may be justifiable both for children using conventional as well as orthodontic pacifiers, although the present data seem to be insufficient to validate any correlation between pacifier sucking and mouth breathing. Therefore, it may be concluded that the pacifier sucking is not strongly related to breathing pattern during the night.

The result of present observational study, reveals that the tongue thrust swallow affected 16.16% of the sample (32 children out of 198), and no significant dependency could be established with pacifier sucking beginning or its duration. In the sample analyzed by Nihi et al., tongue thrust swallow was detected in 27.8% (10 out of 36 subjects), a percentage higher than that of the present study (16.16%; 32 children out of 198) [2]. Nihi et al. associate the tongue thrust swallow with a prolonged pacifier-sucking habit, which delays maturation of the swallowing reflex. The present data suggest that an orthodontic pacifier thin neck nipple structure could be helpful to reduce the occurrence of tongue thrust swallow, as also hypothesized previously [13]. With regard to the poor oral habits, 5.56% of the present sample (11 children out of 198) reported fingersucking/thumbsucking, but no relationship was detected between breastfeeding and fingersucking/thumbsucking. In addition, those children who began to use orthodontic pacifier very early - between 0 and 3 months of life – showed a lower risk to develop fingersucking/thumbsucking habit in respect to children who begin after 4 months (OR 0.13, 95% CI 0.04–0.47, *p* = 0.0004) (Table 3). This result is in an acceptable agreement with the recommendation of both Canadian and American Dental Associations, which suggest pacifiers out of finger/thumb sucking, as it would be more convenient for parents to control the sucking habit, as it is easier to wean a child’s habit from a pacifier, than from a thumb [25, 26].

This finding appears to disagree with what is generally believed for conventional pacifiers, i.e. that if conventional pacifiers are given to infants in the early postpartum period, when they are learning to suck from their mothers’ breasts, its use may act as a favour for a later fingersucking/thumbsucking habit development [27]. In agreement with this statement, Ling et al. reported, from a sample of 1034 Asian children aged 2 to 5 years old, that children who use conventional pacifiers “daily” have significantly higher chances of developing fingersucking/thumbsucking habits [1], while the present study does not confirm this association in the case of orthodontic pacifiers. The present data states the opposite concept, i.e. children who began to use orthodontic pacifier during the first 3 months of life are less likely to develop fingersucking/thumbsucking with respect to children who had begun after 4 months (OR 0.13, 95% CI 0.04–0.47, *p* = 0.0004). It must be noted that Ling et al. report data on conventional pacifiers, and did not analyze the beginning of the pacifier sucking, but only its frequency (a “daily” use or “not daily” use) (OR 2.136; 95% CI 1.11–4.10) [1]. These factors provide satisfactory explanation concerning the different conclusions between the two studies. To the authors best knowledge there is no published work on the relationship between pacifier sucking and fingersucking. The only study performed is the one reported in 1977, the author came to the conclusion that there exists an inverse relation between two habits [28] this is quite compatible with the results obtained in the present study.

Thus, it is not difficult to draw a conclusion that the infants who begin early to use orthodontic pacifiers may experience a kind of satisfaction and this can reduce the urge for further addiction, preventing the development of other poor oral habits.

About the other poor oral habits, the proportions of children having the habits of lip biting, tongue interposition between dental arches, or nail biting were 5.56% (11 children out of 198), 12.63% (25 children out of 198) and 15.15% (30 children out of 198), respectively. In addition, the use of an orthodontic pacifier for more than 2 years seems not to favor the acquisition of additional poor oral habits, even when used for more than 2 years. These percentages appear low and acceptable, if compared with a general population of 1405 preschool children between the ages of 2 and 7 (699 females and 706 males), similar to recently analyzed study in which a percentage of 27.5% of children having bad oral habits was observed. [24]

In addition, no association was detected between the duration or the beginning of orthodontic pacifiers sucking, and the frequencies of these poor oral habits. These data suggest that orthodontic pacifier sucking doesn’t cause the dependency to other poor oral habits. Only a few studies in literature had analyzed the relationship between pacifier sucking and other poor oral habits. About the tongue interposition between dental arches at rest, it was previously investigated in a sample of 36 pre-school children with primary dentition using conventional pacifiers, and it was observed in 38.9% of children (14 subjects out of 36) [2], a percentage higher respect to the present study of 12.63% (25 children out of 198).

In general, the low frequencies of poor oral habits in the present sample could indicate that the use of orthodontic pacifiers do not represent a promoting factor. Despite what reported about conventional pacifiers, it could be hypothesized that infants could experience improved safety and satisfaction due to the unrestricted sucking, as previously reported for breastfeeding [1] and thus no other sucking actions are needed, leading to a low frequency of fingersucking/thumbsucking and other poor oral habits in children using orthodontic pacifiers.

With regard to the prevalence of malocclusions, in the present study, all the various types of malocclusions hypothetically correlated to poor oral habits were summarized together in a unique variable (i.e. “malocclusions”). The logistic regression failed to evidence any correlation between orthodontic pacifier sucking and the presence of malocclusions in the present sample, as only the gender and the breastfeeding variables were significantly related to the prevalence of malocclusion (Table 2). Also, the duration of using orthodontic pacifier was not associated to the prevalence of malocclusions. This could be considered an interesting finding, as malocclusion in the deciduous dentition represents a risk factor for the necessary orthodontic treatment of permanent dentition [29].

In the present sample, 36 children out of 198 (18.18% of the sample) showed a posterior crossbite (28 children showed unilateral crossbite, and 6 children showed bilateral crossbite). The prevalence of posterior crossbite in children using conventional pacifiers varies from 12.8 to 88.9%, as evaluated in a recent systematic review [12]. But in a sample of 55 children using orthodontic pacifiers, Lima et al. [10] reported only 4 cases out of 55 having posterior crossbite (7.27%). To explain the difference with the present sample, we should consider that Lima et al. recorded a very low prevalence of crossbite (6 children out of 55, i.e. less than 10% of the sample) even among children using conventional pacifiers. Thus, the results observed from Lima et al. [10] could be associated not only to the type of pacifier, but probably to the lower mean age of children, that was 28.2 ± 1.9 months (with an initial age range for selection of subjects indicated as 12–24 months) in their sample, while in the present study the age of the children ranged between 3 and 5 years. Furthermore, Lima et al. excluded subjects with enlarged adenoids or respiratory problems, with history of finger sucking, lip sucking, lip biting and lingual interposition, while in the present sample these exclusion criteria were not adopted. Thus, the percentage observed in the present sample appear more generalizable to the population of children adopting orthodontic pacifiers.

From the present data, no significant correlation was found out between the beginning of using orthodontic pacifier, its duration, and the frequency of crossbite. Previous literature based on conventional pacifiers, strongly correlates the posterior crossbite with the duration of the habit, until 4–6 years of age [30], and with the use of a pacifier for more than 36 months [31], more than 2 years [2], more than 15.5 months [6], or more than 1 year [1]. The present observation suggests that the design of the orthodontic pacifier doesn’t promote the occurrence of posterior crossbite, even when used for more than 2 years.

Most of the previous literature state that with a long duration and high frequency of conventional pacifier usage, there is a tendency to hyperfunction of the buccinator muscle, which causes a deficiency in transverse growth of the maxilla and increased frequency of crossbites. Differently, orthodontic pacifiers are designed to avoid hyperfunction of the buccinator muscle. However, some previous studies on conventional pacifiers failed to evidence for an association between posterior crossbite and pacifier sucking. For example, Moimaz et al. could not find any statistically significant difference concerning posterior crossbite between the patients with or without previous usage of a pacifier at 12, 18, and 30 months, except when the posterior crossbite was associated with fingersucking [32–34]. The present data did not reveal any relationship between the orthodontic pacifier sucking and the posterior crossbite, even when associated with fingersucking/thumbsucking. The present findings suggest that the use of an orthodontic pacifier should be not correlated to the prevalence of malocclusions in primary dentition, differently from what reported in literatures concerning the usage of conventional pacifiers.

At the end, this fact could be substantiated that contrary to popular belief regarding pacifier usage, an orthodontic type of pacifier has no effect on neither development of malocclusion, nor poor oral habits, this can be one of the considerable points to make orthodontic pacifier more preferable to the conventional ones.

### Limitations of the study

This observational study was subjected to some limitations. A longitudinal design with an additional follow-up would be useful, especially for monitoring those children that used the pacifier for more than 2 years. Anyway, it should be noted that the present report did not indicate any higher risk of malocclusion for children with more than 2 years of pacifier usage with respect to those children with less prolonged usage, that confounders were reported, and adjustments for non-nutritive sucking habits were performed, trying to avoid biased results. It should also be considered that owing to its design, this study could be susceptible to recall bias. Finally, as parents are unable to monitor their children for 24 h per day, there may be an underestimation of the true level of poor oral habits.

## Conclusions

The use of orthodontic pacifiers does not promote the occurrence of poor oral habits in children with primary dentition, despite its prolonged usage. An early orthodontic pacifier’s usage beginning (0–3 months) seems to be associated with a reduced risk of developing fingersucking/thumbsucking habit. The use of an orthodontic pacifier seems not to be related to the development of malocclusions in primary dentition differently from what previously reported in literature for conventional pacifiers. Further prospective controlled studies are encouraged to confirm what reported in the present study about the relationship between the use of orthodontic pacifiers and the incident of malocclusions and poor oral habits.

## Data Availability

The data that support the findings of this study are available from the University of L’Aquila, but restrictions are applied to the availability of these data, which were used under license for the current study, and so are not publicly available. Data are however available from the authors upon reasonable request and with permission of the University of L’Aquila.

## Abbreviations

95% CI: 95% Confidence interval
CI: Confidence interval
OR: Odds ratio
SPSS: Statistical Package for Social Science
